# Supplementary figures and images for: Aboveground and belowground arthropods experience different relative influences of stochastic versus deterministic community assembly processes following disturbance
Source: PeerJ. 2016 Oct 13;4:e2545. doi: 10.7717/peerj.2545 (PMC5068348; doi:10.7717/peerj.2545)

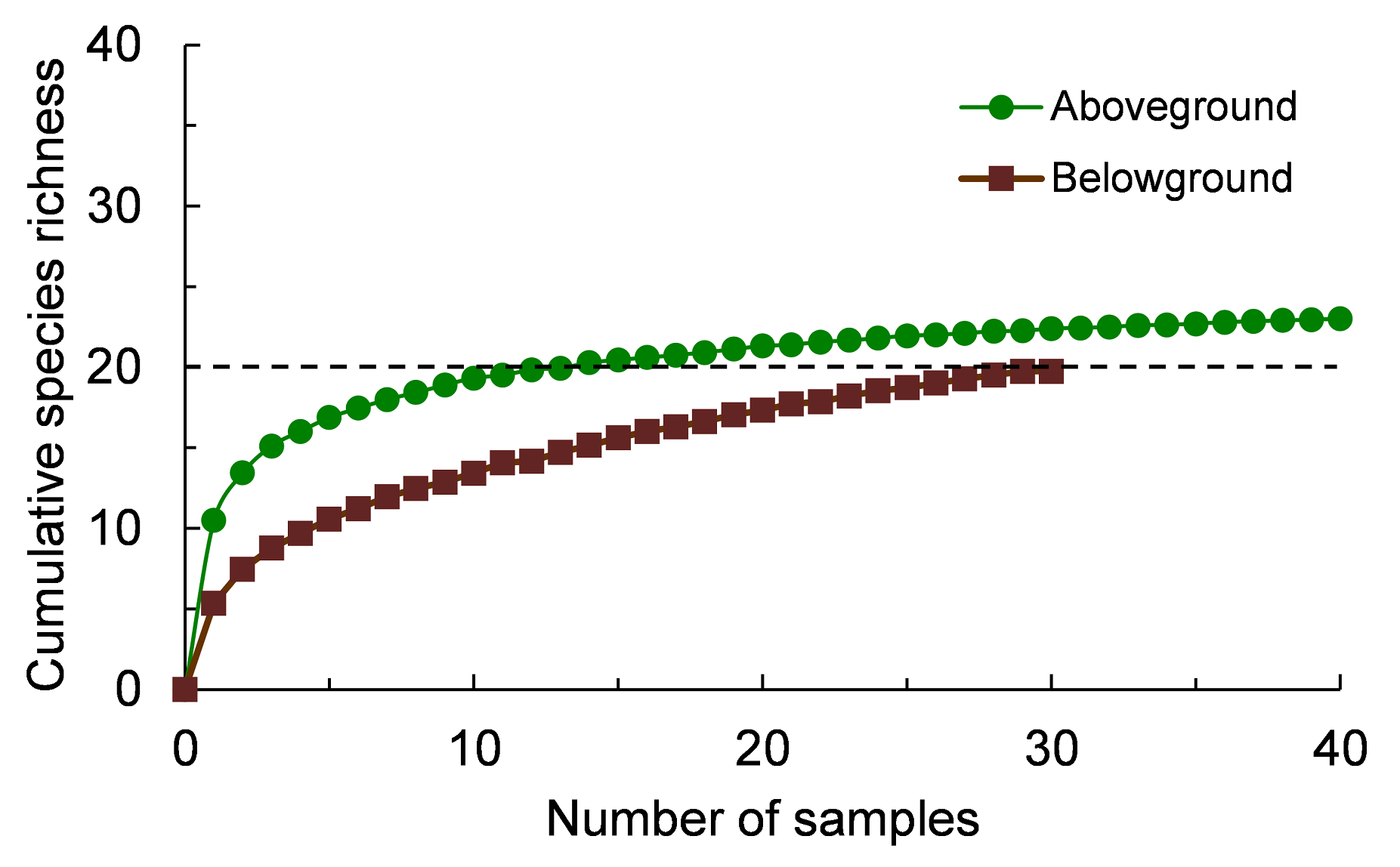

Supplement: Figure S1 — Morphospecies (“species”) accumulation curves for aboveground and belowground arthropod communities sampled along a five-year chronosequence of insect-induced tree mortality in a subalpine conifer forest. Aboveground communities were sampled via pitfall traps which yielded a total of 23 species. Belowground communities were sampled via modified Winkler funnels which yielded a total of 20 species. [file peerj-04-2545-s001.png]
