# Supplementary material for: Aboveground and belowground arthropods experience different relative influences of stochastic versus deterministic community assembly processes following disturbance
Source: PeerJ. 2016 Oct 13;4:e2545. doi: 10.7717/peerj.2545 (PMC5068348; doi:10.7717/peerj.2545)
Supplement: Table S1 [file peerj-04-2545-s002.docx]

| **Supplemental Table 1** Taxonomic classification and proportional abundances of morphospecies captured in aboveground and belowground communities | | | | | |
| --- | --- | --- | --- | --- | --- |
|  |  |  |  |  |  |
| **Class** | **Order** | **Family/subfamily** | **Community** | **% of Community** | **Taxon #** |
| Arachnida | Acari | —— | Belowground | 2.11 | 1 |
| Arachnida | Acari | —— | Belowground | 2.70 | 2 |
| Arachnida | Acari | —— | Above/Belowground | 2.86 | 3 |
| Arachnida | Acari | —— | Belowground | < 1.0 | 4 |
| Arachnida | Acari | —— | Belowground | < 1.0 | 5 |
| Arachnida | Araneae | —— | Aboveground | 2.51 | 6 |
| Arachnida | Araneae | —— | Aboveground | < 1.0 | 7 |
| Arachnida | Araneae | —— | Aboveground | < 1.0 | 8 |
| Arachnida | Araneae | —— | Aboveground | < 1.0 | 9 |
| Arachnida | Araneae | —— | Aboveground | < 1.0 | 10 |
| Arachnida | Araneae | —— | Aboveground | < 1.0 | 11 |
| Arachnida | Araneae | —— | Aboveground | < 1.0 | 12 |
| Arachnida | Araneae | —— | Aboveground | < 1.0 | 13 |
| Arachnida | Araneae | —— | Belowground | < 1.0 | 14 |
| Arachnida | Araneae | —— | Belowground | < 1.0 | 15 |
| Chilopoda | Scolopendromorpha | —— | Belowground | < 1.0 | 16 |
| Diplopoda | Julida | —— | Belowground | < 1.0 | 17 |
| Hexapoda | Coleoptera | Scolytinae | Aboveground | 31.53 | 18 |
| Hexapoda | Coleoptera | Staphylinidae | Belowground | < 1.0 | 19 |
| Hexapoda | Coleoptera | Nitidulidae | Aboveground | < 1.0 | 20 |
| Hexapoda | Coleoptera | Tenebrionidea | Aboveground | < 1.0 | 21 |
| Hexapoda | Coleoptera | Scolytinae | Aboveground | < 1.0 | 22 |
| Hexapoda | Coleoptera | Carabidae | Aboveground | < 1.0 | 23 |
| Hexapoda | Coleoptera | Scarabidae | Aboveground | < 1.0 | 24 |
| Hexapoda | Coleoptera | Unknown (immature) | Belowground | < 1.0 | 25 |
| Hexapoda | Coleoptera | Unknown (immature) | Belowground | < 1.0 | 26 |
| Hexapoda | Collembola | Isotomidae | Above/Belowground | 2.51 | 27 |
| Hexapoda | Collembola | Isotomidae | Above/Belowground | 4.25 | 28 |
| Hexapoda | Collembola | Isotomidae | Belowground | < 1.0 | 29 |
| Hexapoda | Collembola | Isotomidae | Belowground | < 1.0 | 30 |
| Hexapoda | Collembola | Isotomidae | Belowground | < 1.0 | 31 |
| Hexapoda | Homoptera | Aphidae | Aboveground | < 1.0 | 32 |
| Hexapoda | Hymenoptera | Formicidae | Aboveground | 8.09 | 33 |
| Hexapoda | Hymenoptera | Formicidae | Above/Belowground | 10.85 | 34 |
| Hexapoda | Hymenoptera | Formicidae | Aboveground | 25.98 | 35 |
| Hexapoda | Hymenoptera | Formicidae | Aboveground | < 1.0 | 36 |
| Hexapoda | Orthoptera | Acrididae | Aboveground | < 1.0 | 37 |
| Hexapoda | Thysanoptera | Phlaeothripidae | Belowground | < 1.0 | 38 |
| Hexapoda | Thysanoptera | Thripidae | Belowground | < 1.0 | 39 |
