# Supplementary material for: Aboveground and belowground arthropods experience different relative influences of stochastic versus deterministic community assembly processes following disturbance
Source: PeerJ. 2016 Oct 13;4:e2545. doi: 10.7717/peerj.2545 (PMC5068348; doi:10.7717/peerj.2545)
Supplement: Table S2 [file peerj-04-2545-s003.docx]

| **Supplemental Table 2** Mean null deviation values across a 5-year chrono-sequence of tree mortality | | | | |
| --- | --- | --- | --- | --- |
|  |  |  |  |  |
| Group | Chronosequence year | Mean (± 2 S.D.) | | |
| Aboveground | 0 | -0.16 | ± | 0.0012 |
|  | 1 | -0.21 | ± | 0.0029 |
|  | 2 | -0.18 | ± | 0.0021 |
|  | 3 | -0.26 | ± | 0.0017 |
|  | 4 | -0.13 | ± | 0.0015 |
| Belowground | 0 | -0.19 | ± | 0.0008 |
|  | 1 | -0.16 | ± | 0.0035 |
|  | 2 | 0.08 | ± | 0.0042 |
|  | 3 | -0.07 | ± | 0.0009 |
|  | 4 | -0.14 | ± | 0.0388 |
