# Supplementary material for: Aboveground and belowground arthropods experience different relative influences of stochastic versus deterministic community assembly processes following disturbance
Source: PeerJ. 2016 Oct 13;4:e2545. doi: 10.7717/peerj.2545 (PMC5068348; doi:10.7717/peerj.2545)
Supplement: Table S3 [file peerj-04-2545-s004.docx]

| **Supplemental Table 3** Soil chemical and microbial properties associated with arthropod trapping sites across a five year chronosequence of insect induced tree mortality | | | | | | | | | | | | | | |
| --- | --- | --- | --- | --- | --- | --- | --- | --- | --- | --- | --- | --- | --- | --- |
|  |  |  |  |  |  |  |  |  |  |  |  |  |  |  |
| Time since tree mortality (yrs) | Soil moisture | | Total N | | NH_4_^+^ | | Total C | | DOC | | pH | | Micro. biomass | |
|  | (%H_2_O) | | (%) | | (ppm) | | (%) | | (mg/g dry soil) | |  | | (mg C/g dry soil) | |
| 0 | 4.29 | ± (0.58) | 0.11 | ± (0.01) | 0.30 | ± (0.03) | 2.57 | ± (0.29) | 0.06 | ± (0.01) | 6.26 | ± (0.07) | 0.24 | ± (0.02) |
| 1 | 4.59 | ± (1.35) | 0.07 | ± (0.01) | 0.43 | ± (0.11) | 1.85 | ± (0.22) | 0.06 | ± (0.01) | 6.39 | ± (0.11) | 0.19 | ± (0.02) |
| 2 | 5.08 | ± (1.09) | 0.13 | ± (0.03) | 0.61 | ± (0.12) | 2.93 | ± (0.59) | 0.10 | ± (0.03) | 6.34 | ± (0.16) | 0.28 | ± (0.04) |
| 3 | 5.21 | ± (0.68) | 0.08 | ± (0.01) | 0.28 | ± (0.05) | 2.08 | ± (0.25) | 0.06 | ± (0.01) | 6.64 | ± (0.13) | 0.21 | ± (0.02) |
| 4 | 7.01 | ± (1.68) | 0.10 | ± (0.01) | 0.36 | ± (0.10) | 2.61 | ± (0.34) | 0.07 | ± (0.01) | 6.42 | ± (0.13) | 0.26 | ± (0.03) |
| *P*-value | > 0.10 | | 0.06 | | > 0.10 | | > 0.10 | | > 0.10 | | > 0.10 | | > 0.10 | |
| Soil moisture was measured via gravimetric dry-down, total soil N and C were determined by combustion, NH_4_^+^ and DOC were extraced with K_2_SO_4_ , microbial biomass was determined using fumigation proceedures. | | | | | | | | | | | | | | |
|  |  |  |  |  |  |  |  |  |  |  |  |  |  |  |
